# Structure and Electron Mobility of ScN Films Grown on α-Al_2_O_3_(1$\bar{1}$02) Substrates

**DOI:** 10.3390/ma11122449

**Published:** 2018-12-03

**Authors:** Takeshi Ohgaki, Isao Sakaguchi, Naoki Ohashi

**Affiliations:** 1 Electroceramics Group, Research Center for Functional Materials, National Institute for Materials Science, 1-1 Namiki, Tsukuba, Ibaraki 305-0044, Japan; OHASHI.Naoki@nims.go.jp; 2 Ceramics Surface and Interface Group, Research Center for Functional Materials, National Institute for Materials Science, 1-1 Namiki, Tsukuba, Ibaraki 305-0044, Japan; SAKAGUCHI.Isao@nims.go.jp

**Keywords:** scandium nitride, thin film, heterostructure, electric property

## Abstract

Scandium nitride (ScN) films were grown on α-Al_2_O_3_($1\bar{1}02$) substrates using the molecular beam epitaxy method, and the heteroepitaxial growth of ScN on α-Al_2_O_3_($1\bar{1}02$) and their electric properties were studied. Epitaxial ScN films with an orientation relationship (100)ScN || ($1\bar{1}02$)α-Al_2_O_3_ and [001]ScN || [$11\bar{2}0$]α-Al_2_O_3_ were grown on α-Al_2_O_3_($1\bar{1}02$) substrates. Their crystalline orientation anisotropy was found to be small. In addition, [100] of the ScN films were tilted along [$\bar{1}101$] of α-Al_2_O_3_($1\bar{1}02$) in the initial stage of growth. The tilt angle between the film growth direction and [100] of ScN was 1.4–2.0° and increased with growth temperature. The crystallinity of the ScN films also improved with the increasing growth temperature. The film with the highest Hall mobility was obtained at the boundary growth conditions determined by the relationship between the crystallinity and the nonstoichiometric composition because the film with the highest crystallinity was obtained under the Sc-rich growth condition. The decreased Hall mobility with a simultaneous improvement in film crystallinity was caused by the increased carrier scattering by the ionized donors originating from the nonstoichiometric composition.

## 1. Introduction

Scandium nitride (ScN) is a potential semiconductor with a rock-salt structure used to improve the performance of devices based on gallium nitride (GaN) because ScN is lattice-matched to zinc-blende GaN and wurtzite GaN. In the case of the heterostructure of ScN and wurtzite GaN, possible lattice matching of the nitrogen sublattice is expected at (111)ScN||(0001)GaN interfaces. From these expectations, theoretical [1,2,3,4] and experimental [5,6,7,8,9,10] research has been reported on the combination of ScN with GaN-related materials, which achieves the growth of GaN/ScN heterostructures and ScGaN alloys. ScN is mechanically and chemically stable and shows high conductivity without any deliberate doping. The most remarkable feature of ScN is its high electron mobility. Dismukes et al. reported that the Hall mobility (*μ*) of hydride vapor-phase-epitaxy-grown ScN films is approximately 150 cm^2^ V^−1^ s^−1^ even though its carrier concentration (*n*) is 1 × 10^20^ cm^−3^ at room temperature [11]. Assuming that carrier scattering by the ionized donor is the dominant scattering process, a higher electron *μ* in a lightly doped ScN is expected.

ScN has so far been prepared by various film growth processes to examine semiconducting properties. Although lattice-matched substrates are essentially suitable for the epitaxial growth of a high-quality single-crystal film, magnesium oxide (MgO) [12,13,14,15,16,17,18,19,20,21] or α-Al_2_O_3_ [11,12,22,23,24,25] substrates have been used likely because of the lack of lattice-matched single crystals. MgO has the same crystal structure as ScN (i.e., rock-salt type structure), and the lattice mismatch between ScN and MgO is +6.9%. In contrast, α-Al_2_O_3_ has a corundum-type structure, and the heterostructures of ScN and α-Al_2_O_3_ are complicated. For example, epitaxial ScN films with an orientation relationship of (110)ScN || ($10\bar{1}0$)α-Al_2_O_3_ and [001]ScN || [$1\bar{2}10$]α-Al_2_O_3_ were grown on α-Al_2_O_3_($10\bar{1}0$) substrates [22,24]. In this case, the epitaxial relationship had −2.0% and −5.5% lattice mismatch along the 0001 and $1\bar{2}10$ directions, respectively. As for the ScN films grown on α-Al_2_O_3_($1\bar{1}02$), several kinds of crystalline orientations have been reported [11,12,24,25]. Febvrier et al. recently reported the effects of crystalline quality and impurities on the electrical properties of ScN films using MgO(111), α-Al_2_O_3_(0001), and α-Al_2_O_3_($1\bar{1}02$) substrates prepared by the DC magnetron sputtering method [12].

By considering the relationship between *n* and *μ*, which depends on *n* caused by the carrier scattering induced by ionized donors, ScN films with high electron *μ* were obtained using α-Al_2_O_3_($1\bar{1}02$) substrates [11,25]. In general, *μ* of semiconductor films strongly depends on its crystallinity and degree of carrier scattering by ionized impurity. Recent research results indicate that *n* and *μ* of ScN films are strongly affected by growth conditions, especially growth temperature and Sc/N supply ratio, during film growth [13,18,22]. However, no study has yet examined the relationship between crystalline quality and electron *μ* of ScN films grown on α-Al_2_O_3_($1\bar{1}02$) substrates.

In this study, ScN films were grown on α-Al_2_O_3_($1\bar{1}02$) substrates using molecular beam epitaxy (MBE) with radical irradiation to examine the relationship between the crystalline quality and the electrical properties of the films while applying various growth conditions (i.e., substrate temperature and Sc/N supply ratio). The heteroepitaxial structure of ScN on α-Al_2_O_3_($1\bar{1}02$) and the factors determining the electron *μ* of ScN films were investigated.

## 2. Materials and Methods

We used a metal source MBE method using an RF (13.56 MHz) radical source as the nitrogen source. α-Al_2_O_3_ single crystals with ($1\bar{1}02$) faces were selected as substrates for the ScN film growth. Before starting the film growth, the substrates were heated in a growth chamber at 900 °C for 30 min to obtain a clean and highly crystallized surface. Nitrogen plasma generated by an RF radical source was irradiated on the film surface during the film growth. Nitrogen gas (99.99995%) was introduced into the discharge cell. The pressure in the growth chamber was 5.0–5.5 × 10^−6^ Torr. The flow rate and the RF power were 0.7 sccm and 400 W, respectively. A Knudsen cell (K-cell) with a boron nitride crucible was used to supply the Sc (99.9%) flux. The K-cell temperature (*T_Sc_*) was varied in the range of 1300–1325 °C to investigate the effects of the Sc/N supply ratio on the crystalline quality and electrical properties of the films. The film growth temperature (*T_g_*) was also varied between 750 °C and 900 °C to examine the effects of substrate temperature. The film thickness of the films was in the range of 140–300 nm.

The structure and the crystallinity of the films were analyzed using X-ray diffraction (XRD) (X’ Pert MRD, Malvern Panalytical Ltd., Worcestershire, UK) measurements. The θ–2θ, rocking curve (ω-scan), pole figure, and reciprocal space mapping (RSM) modes of the XRD were measured. The measurements in the pole figure and the RSM mode were performed by fixing the sample along the crystal orientation of the α-Al_2_O_3_($1\bar{1}02$) substrate. The cross-sectional images of the ScN films were observed by transmission electron microscopy (TEM) (H-9000UHR, Hitachi High-Technologies Corp., Tokyo, Japan). The morphologies of the film and the substrate surfaces were evaluated by atomic force microscopy (AFM) (SPA-400, SII Nanotechnology Inc., Chiba, Japan). The thicknesses of the films were measured by a surface profiler (Dektak 3030, Sloan Technology Corp., Santa Barbara, CA, USA). The electrical resistivity (*ρ*), *n*, and *μ* of the films were determined by a standard van der Pauw four-probe geometry (8 × 8 mm^2^ films) using Hall effect measurements under a magnetic field of 0.5 T at room temperature (ResiTest 8200, TOYO Corp., Tokyo, Japan). Aluminum electrical contacts were formed using the vacuum evaporation technique. The optical properties of the films were evaluated using regular transmittance measurement (SolidSpec-3700, Shimadzu Corp., Kyoto, Japan).

## 3. Results and Discussion

### 3.1. Heterostructure and Electrical Properties

The AFM measurement revealed that the surface morphologies of the ScN films grown on α-Al_2_O_3_($1\bar{1}02$) were similar to those of the (100)-oriented ScN films grown on MgO (100) substrates, as reported in a previous study [13]. The films grown under *T_Sc_* = 1300 °C at 750 °C ≤ *T_g_* ≤ 900 °C had square grains, while those grown under 1320 ≤ *T_Sc_* ≤ 1325 at *T_g_* = 900 °C had round grains.

The 200 diffraction peaks of ScN were only found with the $1\bar{1}02$ and $2\bar{2}04$ diffraction peaks of the α-Al_2_O_3_($1\bar{1}02$) substrate in the XRD profiles of the θ–2θ scanning mode, and no asymmetry peaks and peak shift were observed on all samples.

**Figure 1:**
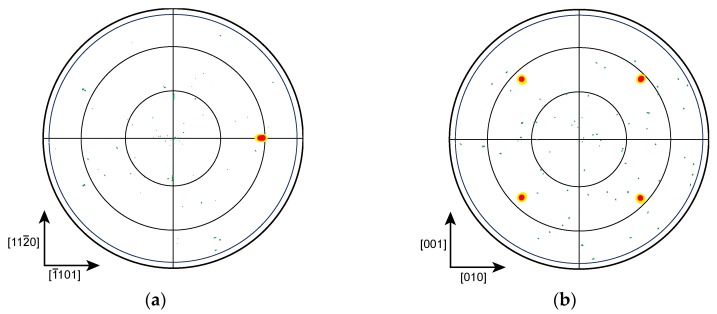
Typical X-ray pole figure pattern of the scandium nitride (ScN) films grown on the α-Al_2_O_3_($1\bar{1}02$) substrates: (**a**) (0006) diffraction of α-Al_2_O_3_($1\bar{1}02$) and (**b**) (222) diffraction of ScN.

Figure 1 shows the typical XRD pole figure patterns of the α-Al_2_O_3_($1\bar{1}02$) substrate and the grown film. One diffraction spot attributed to the α-Al_2_O_3_(0006) and four diffraction spots attributed to the ScN(222) were observed. All the ScN films grown on the α-Al_2_O_3_($1\bar{1}02$) substrates herein showed the same diffraction pattern. The results illustrated that the epitaxial ScN films were grown on the α-Al_2_O_3_($1\bar{1}02$) substrates in spite of different crystal structures (i.e., rock-salt-type and corundum-type structures). The epitaxial relationship between ScN and α-Al_2_O_3_ was (100)ScN || ($1\bar{1}02$)α-Al_2_O_3_ and [001]ScN || [$11\bar{2}0$]α-Al_2_O_3_. Moreover, the four diffraction spots attributed to ScN(222) were slightly located in [$\bar{1}101$] of the α-Al_2_O_3_($1\bar{1}02$) substrates.

**Figure 2:**
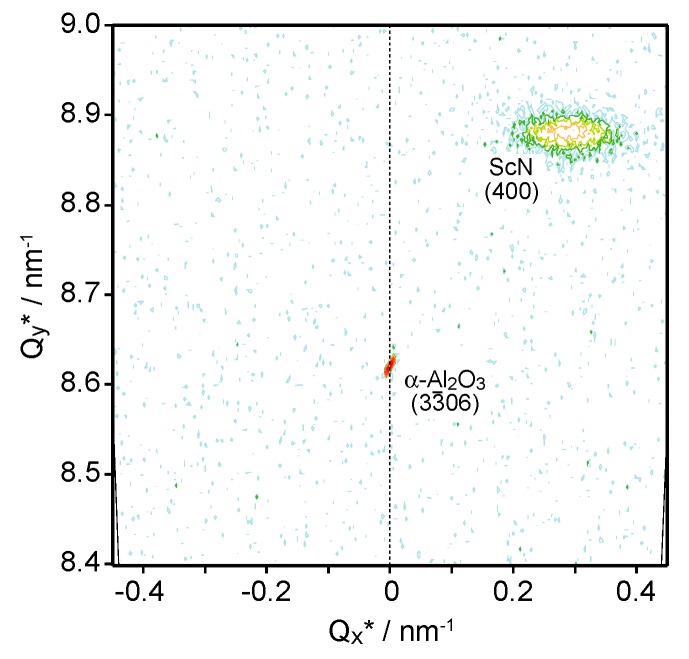
Reciprocal space mapping for the ($3\bar{3}06$) diffraction of the α-Al_2_O_3_($1\bar{1}02$) substrate and the (400) diffraction of the ScN film. The directions of Q_x_* and Q_y_* indicate the $\bar{1}101$ direction of the α-Al_2_O_3_($1\bar{1}02$) substrate and the film growth direction, respectively. The reciprocal lattice point of α-Al_2_O_3_($3\bar{3}06$) is fixed at Q_x_* = 0.

The RSM for the (400) diffraction of the ScN films revealed details of the ScN/α-Al_2_O_3_($1\bar{1}02$) heterostructure. Figure 2 shows the RSM results for the (400) diffraction of the ScN films and the ($3\bar{3}06$) diffraction of the α-Al_2_O_3_($1\bar{1}02$) substrate. The directions of Q_x_* and Q_y_* indicated the $\bar{1}101$ direction of the α-Al_2_O_3_($1\bar{1}02$) substrate and the film growth direction (normal direction of α-Al_2_O_3_($1\bar{1}02$)), respectively. The reciprocal lattice point of α-Al_2_O_3_($3\bar{3}06$) was fixed at Q_x_* = 0. The reciprocal lattice point of ScN(400) was positioned at Q_x_* = 0.3, indicating that [100] of ScN and the film-growth direction were not perfectly parallel. This tilt growth was confirmed in all ScN films on the α-Al_2_O_3_($1\bar{1}02$) substrates.

**Figure 3:**
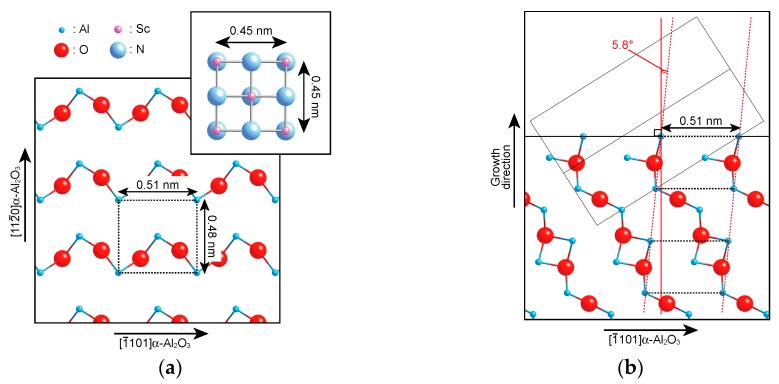
Atomic arrangements of the α-Al_2_O_3_($1\bar{1}02$) substrate and the ScN film: (**a**) α-Al_2_O_3_($1\bar{1}02$) and ScN(100) surfaces and (**b**) cross-section of α-Al_2_O_3_($1\bar{1}02$). The rectangle in (**b**) indicates the unit cell of α-Al_2_O_3_.

The substrate surface heated at 900 °C for 30 min, which was the thermal cleaning condition before the film growth, was observed by AFM to examine the tilt growth origin of the epitaxial films. However, no obvious changes in the surface morphologies, such as step-and-terrace structure and dissymmetric shapes, were observed. Therefore, the reason for the tilt growth seems to be the atomic arraignment of the α-Al_2_O_3_($1\bar{1}02$) substrate. Figure 3a shows the atomic arrangements and the atomic distance of the α-Al_2_O_3_($1\bar{1}02$) and ScN(100) surfaces. The dotted line indicates the suitable epitaxial arrangement between the (100)-oriented ScN film and the α-Al_2_O_3_($1\bar{1}02$) substrate. This epitaxial relationship can be explained as follows: The (100)-oriented ScN has a square unit cell with dimensions of 0.45 nm × 0.45 nm, and the α-Al_2_O_3_($1\bar{1}02$) surface has a rectangular atomic arrangement of Al atoms with dimensions of 0.51 nm × 0.48 nm. In this epitaxial arraignment, the epitaxial relationship shows −12.3% and −5.5% lattice mismatch along the $\bar{1}101$ and $11\bar{2}0$ directions of α-Al_2_O_3_, respectively. As for the cross-sectional atomic arrangements of the α-Al_2_O_3_($1\bar{1}02$) substrate in Figure 3b, the periodic rectangular layers of α-Al_2_O_3_($1\bar{1}02$) in Figure 3a were laminated with shifting toward the $\bar{1}101$ direction of α-Al_2_O_3_($1\bar{1}02$). The angle formed by the normal to the ($1\bar{1}02$) surface and the laminated direction of the periodic rectangular in each laminated layer is 5.8°. Therefore, the atomic arrangement of the α-Al_2_O_3_($1\bar{1}02$) surface layer and the laminated direction of the periodic rectangular of α-Al_2_O_3_($1\bar{1}02$) seemed to cause the tilted growth of the (100)-oriented ScN films on α-Al_2_O_3_($1\bar{1}02$) substrates.

**Figure 4:**
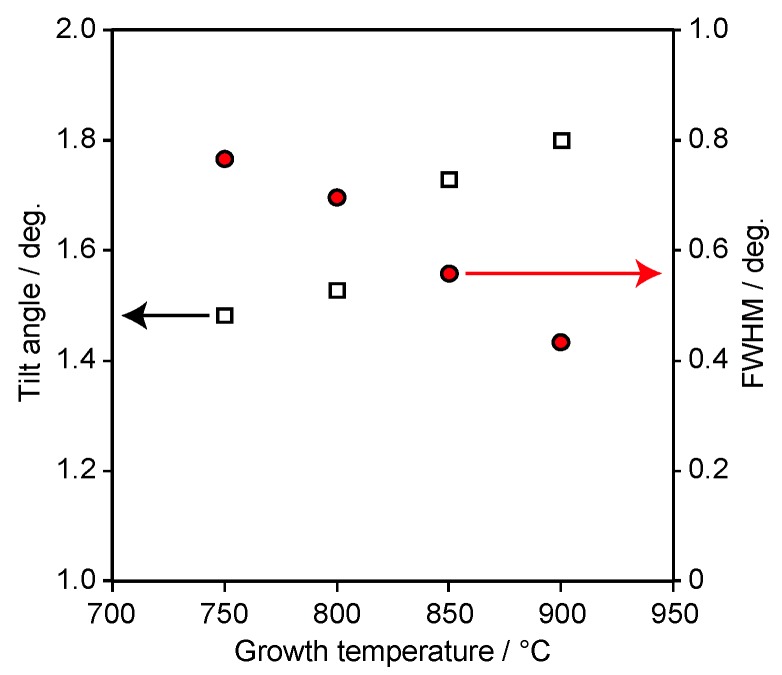
Tilt angle and full width at half-maximum (FWHM) of the X-ray diffraction (XRD) ω-scan for ScN(200) as functions of the growth temperature. The open squares indicate the tilt angle of the ScN films. The red circles indicate the FWHM of the XRD ω-scan for ScN(200). The Sc source temperature is set to 1300 °C. The film’s thickness is in the range of 140–175 nm.

We evaluated the tilt angle and the full width at half-maximum (FWHM) of the ScN(200) diffraction by XRD measurement in ω-scan mode along the $\bar{1}101$ direction of α-Al_2_O_3_ to examine the crystalline orientation and film crystallinity as a function of *T_g_* (Figure 4). The ScN films herein were grown under *T_Sc_* = 1300 °C. The tilt angle between [100] of ScN and the film growth direction indicated by open squares increased with the increasing *T_g_* and was approximately 1.4–2.0°. Therefore, the degree of tilt angle was determined by the film growth conditions and influenced by two growth mechanisms (i.e., one is the non-tilt growth attributed to the atomic arrangement of the α-Al_2_O_3_($1\bar{1}02$) surface layer and the other is the tilt growth attributed to the cross-sectional atomic arrangement of the laminated layer in the α-Al_2_O_3_($1\bar{1}02$) substrate). The FWHM of ScN(200) decreased with the increasing growth temperature, resulting in the improvement of the film crystallinity in the case of high-temperature growth. This improvement was the same as for the MBE-grown ScN films on the MgO(100) [13] and α-Al_2_O_3_($10\bar{1}0$) [22] substrates and was also observed in the ScN films grown using DC magnetron sputtering [12].

**Figure 5:**
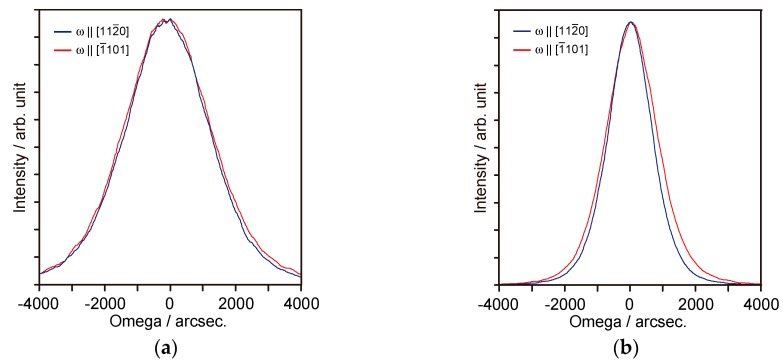
XRD ω-scan profiles for ScN(200) grown on the α-Al_2_O_3_($1\bar{1}02$) substrates: (**a**) ScN films grown at 750 °C and (**b**) ScN films grown at 900 °C. The Sc source temperature is set to 1300 °C. The film thicknesses are in the range of 140–175 nm.

Figure 5 depicts the XRD ω-scan profiles for the (200) diffraction of the ScN films grown at *T_g_* = 750 °C and *T_g_* = 900 °C. The measurements were conducted along [$11\bar{2}0$] and [$\bar{1}101$] of the α-Al_2_O_3_($1\bar{1}02$) substrates. Figure 5 shows that the crystallinity of the films grown at *T_g_* = 900 °C (Figure 5b) was significantly superior to that of the film grown at *T_g_* = 750 °C (Figure 5a). In contrast, the crystalline orientation anisotropy for both films was small, while the crystallinity in the $\bar{1}101$ direction was slightly inferior to that in the $11\bar{2}0$ direction. As mentioned earlier, the lattice mismatch values along the $11\bar{2}0$ and $\bar{1}101$ directions of α-Al_2_O_3_ were −5.5% and −12.3%, respectively. Moreover, the *T_g_* dependency of crystalline anisotropy was clearly observed in the grown films on the α-Al_2_O_3_($10\bar{1}0$) substrates [22]. In this case, the epitaxial relationship had −2.0% and 5.5% lattice mismatch along [0001] and [$1\bar{2}10$] of α-Al_2_O_3_($10\bar{1}0$), respectively. Therefore, the tilt growth is considered to be effective in relaxing the restriction of the substrate lattice caused by the large lattice mismatch along the $\bar{1}101$ direction of the α-Al_2_O_3_ substrate.

**Figure 6:**
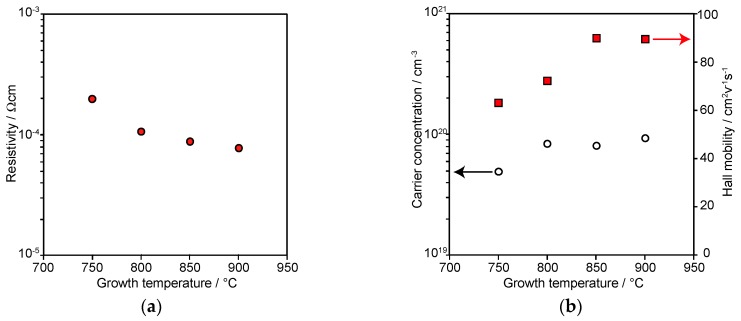
Growth temperature dependence of (**a**) resistivity, (**b**) carrier concentration, and Hall mobility of the ScN film. The red circles indicate resistivity. The open circles denote the carrier concentration. The red squares depict the Hall mobility of the films. The Sc source temperature is set to 1300 °C. The film thicknesses are in the range of 140–175 nm.

The Hall effect measurement was performed at room temperature to clarify the electrical properties of the ScN films grown on the α-Al_2_O_3_($1\bar{1}02$) substrates (*T_Sc_* = 1300 °C). All the epitaxial ScN films grown on the α-Al_2_O_3_($1\bar{1}02$) substrates showed n-type conduction. Electric orientation anisotropy was not observed in spite of the large anisotropic lattice mismatch. The *ρ* of ScN films shown in Figure 6a decreased with the increasing *T_g_*. The values were approximately 10^−4^ Ω cm. Figure 6b shows the *T_g_* dependence of *n* and *μ* of the ScN film. The open circles indicate *n*, while the red squares represent *μ* of the films. The *n* values of the films grown on the α-Al_2_O_3_($1\bar{1}02$) substrates were approximately 10^20^ cm^−3^, while the *μ* values were in the ranges of 60–100 cm^2^ V^−1^ s^−1^. The *μ* values of the films increased with *T_g_*. The increase of *μ* with the increasing *T_g_* of the films grown on α-Al_2_O_3_($1\bar{1}02$) was likely caused by the improvement in the film crystallinity, as shown in Figure 4. A similar *T_g_* dependence of the film crystallinity and *μ* of the films were observed in the ScN film growth on the MgO(100) [13] and α-Al_2_O_3_($10\bar{1}0$) [22] substrates. In contrast, *n* of the films were nearly independent of *T_g_*, indicating that the native donor concentrations, such as N vacancies and Sc at interstitial sites, were almost the same in the range of 750 °C ≤ *T_g_* ≤ 900 °C.

### 3.2. Effects of the Sc/N Supply Ratio

The ScN films were grown under *T_Sc_* = 1320, 1323, and 1325 °C to examine the effects of the Sc/N supply ratio on the crystalline quality and electrical properties of the films. The film color changed from an orange-brown color to a dark-brown color with the increasing *T_Sc_*. The transmission spectra of these films were consistent with those of the previously reported data by Smith et al. [18].

**Figure 7:**
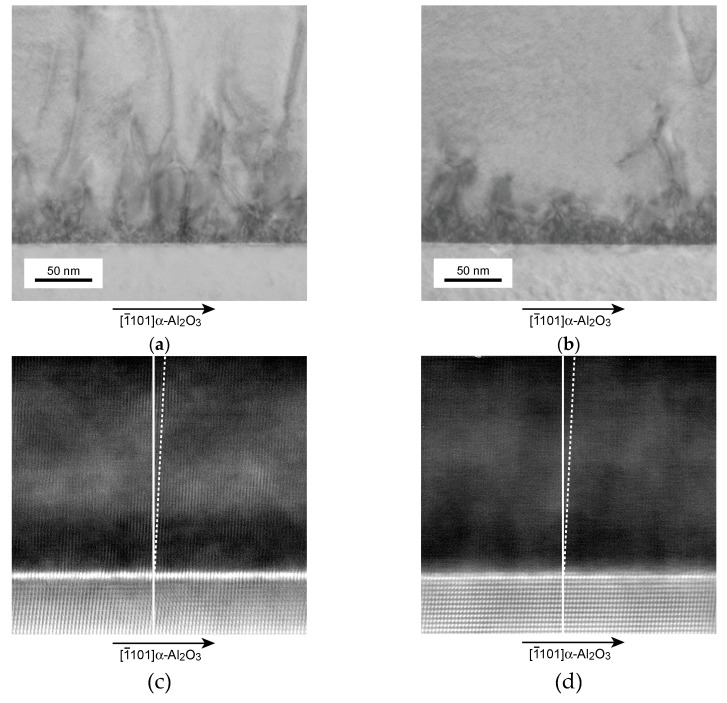
Transmission electron microscopy (TEM) and magnified images of the interface between the ScN films and α-Al_2_O_3_ grown under *T_Sc_* = 1320 °C (**a**,**c**) and *T_Sc_* = 1325 °C (**b**,**d**). The white lines indicate the film growth direction. The dotted lines depict [100] of ScN. Both films are grown at 900 °C.

Figure 7 compares the cross-sectional TEM images of the films and the magnified images of the interface between the films and the substrate grown under *T_Sc_* = 1320 °C (Figure 7a,c) and *T_Sc_* = 1325 °C (Figure 7b,d). These films were grown at *T_g_* = 900 °C. The film thicknesses were in the range of 230–300 nm. The white lines in Figure 7c,d indicate the film growth direction, while the dotted lines indicate [100] of ScN. First, we compared the effect of *T_Sc_* on the structure and dislocations of the films. The magnified images revealed that the tilt growth of the films occurred at the initial growth stage. The angles of both films estimated from the atomic arrangement were approximately 2.0°. This result agreed with the XRD measurement results shown in Figure 2 and Figure 4. The dislocations of both films decreased as the film thickness increased. The dislocation density at the film surface region in the films grown under *T_Sc_* = 1325 °C was clearly less than that in the films grown at *T_Sc_* = 1320 °C, although a high dislocation density was found in the ScN grown under *T_Sc_* = 1325 °C near the interfacial region. The high dislocation density near the interface region clearly contributed to the relaxation of restriction from the substrate, resulting in the decrease of the dislocation in the film surface and middle region, which was also the reason why the increase of the Sc flux during film growth improved the crystallinity of the ScN films. The FWHM values of the XRD ω-scan for the ScN(200) grown at *T_Sc_* = 1320 °C and *T_Sc_* = 1325 °C were 0.41° and 0.16°, respectively. These results also indicated that the film crystallinity was strongly affected by the Sc/N supply ratio, and the high-*T_Sc_* growth condition was effective in realizing high-crystalline quality films. The similar relation between the crystallinity and the flux supply ratio of the source materials during growth was reported in ZnO and ScN prepared by the MBE method and could be explained by the qualitative change in the surface diffusion of the adatoms. For growth using the MBE method, a high diffusion length could be generally achieved by high *T_g_* and a suitable flux ratio [18,26].

The electric properties of the ScN films were affected by the Sc/N supply ratio and the film crystalline quality of the ScN films. The *n* and *μ* values of the films grown under *T_Sc_* = 1320 °C were 3.2 × 10^20^ cm^−3^ and 143 cm^2^ V^−1^ s^−1^, respectively (*ρ* = 7.8 × 10^−5^ Ω cm; thickness = 230 nm). In contrast, the *n* and *μ* values of the films grown under *T_Sc_* = 1325 °C were 1.0 × 10^21^ cm^−3^ and 147 cm^2^ V^−1^ s^−1^, respectively (*ρ* = 4.2 × 10^−5^ Ω cm; thickness = 300 nm). Both films had almost the same *μ* values, while *n* for films grown under *T_Sc_* = 1325 °C was approximately three times as large as that of the film grown under *T_Sc_* = 1320 °C. In the present study, the film with the highest *μ* value was obtained by applying *T_Sc_* = 1322 °C (thickness = 250 nm). The *n* and *μ* values were 7.3 × 10^20^ cm^−3^ and 181 cm^2^ V^−1^ s^−1^, respectively (*ρ*= 4.7 × 10^−5^ Ω cm; FWHM of XRD ω-scan = 0.19°).

We assumed that the boundary temperature existed (*T_Sc_* = 1322 °C) in terms of the electric properties of the films. The *T_Sc_* dependence of *n* and *μ* of the films could likely be categorized into two groups. The Sc-rich growth condition indicated that *T_Sc_* > 1322 °C, while the N-rich growth condition presented that the *T_Sc_* < 1322 °C. A decrease in *μ* and an increase in *n* occurred for the film grown under the Sc-rich growth condition in comparison with the films grown under *T_Sc_* = 1322 °C. The decrease in *μ* with the increase in *n* was caused by the increased carrier scattering by the native shallow donors in ScN. The native defect concentration corresponding to the nonstoichiometric composition was increased by the Sc-rich growth conditions. The native defects increased with *n* and decreased with *μ*. Similar behaviors were found in the ScN films grown on MgO(100) [13,18] and α-Al_2_O_3_($10\bar{1}0$) [22] substrates. By contrast, the results of the increase in *μ* of the films grown under *T_Sc_* = 1322 °C in comparison with the films grown under the N-rich growth condition indicated that *μ* of the film was correlated with the film crystallinity. As shown in Figure 4 and Figure 6 (N-rich growth condition), both improvement in crystallinity and increase in *μ* of the films occurred with the increasing *T_g_*. Therefore, high-*μ* films could be obtained at the boundary temperature of *T_Sc_* = 1322 °C.

Thus, the study results revealed that the *μ* values of the ScN films were strongly influenced by their crystalline quality and nonstoichiometric composition, which could be controlled by varying the Sc/N supply ratio during film growth. The ScN films with high crystalline quality could be grown by applying Sc-rich growth conditions; however, those with high *μ* could be grown at the boundary temperature determined by the relationship between crystallinity and nonstoichiometric composition.

## 4. Conclusions

Epitaxial ScN films were grown herein on α-Al_2_O_3_($1\bar{1}02$) substrates by an MBE with the radical irradiation method. The epitaxial relation between ScN and α-Al_2_O_3_ was (100)ScN || ($1\bar{1}02$)α-Al_2_O_3_ and [001]ScN || [$11\bar{2}0$]α-Al_2_O_3_, and [100] of the films was slightly tilted along [$\bar{1}101$] of α-Al_2_O_3_($1\bar{1}02$) from the initial stage of film growth. The tilt angle between [100] of ScN and the film growth direction was 1.4–2.0° and depended on the growth temperature. Crystalline orientation anisotropy of the ScN films was small despite the large lattice mismatch between ScN and α-Al_2_O_3_($1\bar{1}02$). The crystallinity of the ScN films improved with the increasing growth temperature and Sc-cell temperature. The film with the highest Hall mobility was grown at the boundary growth conditions determined by the relationship between crystallinity and nonstoichiometric composition. The decrease in the Hall mobility with the simultaneous improvement in the film crystallinity in the Sc-rich growth condition was caused by the increased carrier scattering, which consequently arose from the ionized donors caused by the nonstoichiometric composition of ScN.

## References

[1] Alsaad A., Ahmad A., Alta’ani H., Alshyab R. (2008). A first-principles-derived method for computing the piezoelectric coefficients of complex semiconductor Sc_1−x_Ga_x_N alloys. Physica B.

[2] Zerroug S., Sahrauoui F.A., Bouarissa N. (2008). Ab initio calculations of structural properties of Sc_x_Ga_1−x_N. J. Appl. Phys..

[3] Ranjan V., Bin-Omran S., Sichuga D., Nichols R.S., Bellaiche L., Alsaad A. (2005). Properties of GaN/ScN and InN/ScN superlattices from first principles. Phys. Rev. B.

[4] Fredj A.B., Oussaifi Y., Bouarissa N., Said M. (2006). Electronic properties of zinc-blende Sc_x_Ga_1−x_N. Phys. Status Solidi B.

[5] Hall J.L., Moram M.A., Sanchez A., Novikof S.V., Kent A.J., Foxon C.T., Humphreys C.J., Campion R.P. (2009). Growth of ScN epitaxial films by plasma-assisted molecular beam epitaxy. J. Cryst. Growth.

[6] Moram M.A., Zhang Y., Joyce T.B., Holec D., Chalker P.R., Mayrhofer P.H., Kappers M.J., Humphreys C. (2009). Structural properties of wurtzite-like ScGaN films grown by NH_3_-molecular beam epitaxy. J. Appl. Phys..

[7] Moram M.A., Zhang Y., Kappers M.J., Barber Z.H., Humphreys C.J. (2007). Dislocation reduction in gallium nitride films using scandium nitride interlayers. Appl. Phys. Lett..

[8] Höglund C., Bareño J., Birch J., Alling B., Czigány Z., Hultman L. (2009). Cubic Sc_1−x_Al_x_N solid solution thin films deposited by reactive magnetron sputter epitaxy onto ScN(111). J. Appl. Phys..

[9] Perjeru F., Bai X., Ortiz-Libreros M.I., Higgins R., Kordesch M.E. (2001). ScN/GaN heterojunctions: Fabrication and characterization. Appl. Surf. Sci..

[10] Little M.E., Kordesch M.E. (2001). Band-gap engineering in sputter-deposited Sc_x_Ga_1−x_N. Appl. Phys. Lett..

[11] Dismukes J.P., Yim W.M., Ban V.S. (1972). Epitaxial growth and properties of semiconducting ScN. J. Cryst. Growth.

[12] Febvrier A.L., Tureson N., Stilkerich N., Greczynski G., Eklund P. (2019). Effect of impurities on morphology, growth mode, and thermoelectric properties of (111) and (001) epitaxial-like ScN films. J. Phys. D.

[13] Ohgaki T., Watanabe K., Adachi Y., Sakaguchi I., Hishita S., Ohashi N., Haneda H. (2013). Electrical properties of scandium nitride epitaxial films grown on (100) magnesium oxide substrates by molecular beam epitaxy. J. Appl. Phys..

[14] Al-Brithen H.A.H., Trifan E.M., Ingram D.C., Smith A.R., Gall D. (2002). Phase stability, nitrogen vacancies, growth mode, and surface structure of ScN(001) under Sc-rich conditions. J. Cryst. Growth.

[15] Al-Brithen H., Smith A.R. (2000). Molecular beam epitaxial growth of atomically smooth scandium nitride films. Appl. Phys. Lett..

[16] Al-Brithen H.A., Smith A.R., Gall D. (2004). Surface and bulk electronic structure of ScN(001) investigated by scanning tunneling microscopy/spectroscopy and optical absorption spectroscopy. Phys. Rev. B.

[17] Gall D., Petrov I., Madsen L.E., Sundgren J.E., Greene J.E. (1998). Microstructure and electronic properties of the refractory semiconductor ScN grown on MgO(001) by ultra-high-vacuum reactive magnetron sputter deposition. J. Vac. Sci. Technol. A.

[18] Smith A.R., Al-Brithen H.A.H., Ingram D.C., Gall D. (2001). Molecular beam epitaxy control of the structural, optical, and electronic properties of ScN(001). J. Appl. Phys..

[19] Burmistrova P.V., Maassen J., Favaloro T., Saha B., Salamat S., Koh Y.R., Lundstrom M.S., Shakouri A., Sands T.D. (2013). Thermoelectric properties of epitaxial ScN films deposited by reactive magnetron sputtering onto MgO(001) substrates. J. Appl. Phys..

[20] Deng R., Ozsdolay B.D., Zheng P.Y., Khare S.V., Gall D. (2015). Optical and transport measurement and first-principles determination of the ScN band gap. Phys. Rev. B.

[21] Gall D., Petrov I., Hellgren N., Hultman L., Sundgren J.E., Greene J.E. (1998). Growth of poly- and single-crystal ScN on MgO(001): Role of low-energy N_2_^+^ irradiation in determining texture, microstructure evolution, and mechanical properties. J. Appl. Phys..

[22] Ohgaki T., Sakaguchi I., Ohashi N., Haneda H. (2017). Heteroepitaxial growth and electric properties of (110)-oriented scandium nitride films. J. Cryst. Growth.

[23] Kerdsongpanya S., Nong N.V., Pryds N., Žukauskaite A., Jensen J., Birch J., Lu J., Hultman L., Wingqvist G., Eklund P. (2011). Anomalously high thermoelectric power factor in epitaxial ScN thin films. Appl. Phys. Lett..

[24] Oshima Y., Villora E.G., Shimamura K. (2014). Hydride vapor phase epitaxy and characterization of high-quality ScN epilayers. J. Appl. Phys..

[25] Gregoire G.M., Kirby S.D., Scopelianos G.E., Lee F.H., van Dover R.B. (2008). High mobility single crystalline ScN and single-orientation epitaxial YN on sapphire via magnetron sputtering. J. Appl. Phys..

[26] Ohgaki T., Ohashi N., Kakemoto H., Wada S., Adachi Y., Haneda H., Tsurumi T. (2003). Growth condition dependence of morphology and electric properties of ZnO films on sapphire substrates prepared by molecular beam epitaxy. J. Appl. Phys..

